# Phosphoproteomic analysis in a mouse model reveals ERK signaling as a key modulator of inflammatory response in nasal mucosa associated with childhood allergic rhinitis

**DOI:** 10.3389/fimmu.2026.1795749

**Published:** 2026-08-27

**Authors:** Xiaojun Zhan, Mengyao Li, Siyu Ye, Ruikun Wang, Shengchen Zhao, Changhao Xie, Weijing Wang, Shan Wang, Qinglong Gu

**Affiliations:** 1 Department of Otolaryngology-Head and Neck Surgery, Allergy Center, Capital Center for Children’s Healthy, Capital Medical University, Capital Institute of Pediatrics, Beijing, China; 2 Department of Bacteriology, Capital Institute of Pediatrics, Beijing, China; 3 Capital Institute of Pediatrics-Peking University Teaching Hospital, Beijing, China; 4 Graduate School of Peking Union Medical College, Beijing, China; 5 Beijing Municipal Key Laboratory of Child Development and Nutriomics, Capital Institute of Pediatrics, Beijing, China

**Keywords:** allergic rhinitis, ERK, inflammatory response, nasal mucosa, phosphorylation

## Abstract

Childhood allergic rhinitis (AR) is a multifactorial condition arising from the interplay between genetic predisposition and environmental exposures. Although protein phosphorylation is widely recognized as a key regulator of gene expression across various physiological and pathological states, its global alterations in the nasal mucosa of pediatric patients with AR and their subsequent impact on mucosal function and inflammatory pathways remain incompletely characterized. Our study aimed to elucidate the molecular mechanisms underlying nasal mucosa dysfunction induced by pediatric AR. Our analysis revealed 3,861 proteins encompassing a total of 15,491 phosphorylation sites. Specifically, we detected 441 downregulated phosphorylation sites on 584 proteins and 531 upregulated phosphorylation sites on 722 proteins in the nasal mucosa of the AR group. Our proteomics findings suggest that the dysregulation of immune activation and metabolic regulation may contribute to AR pathophysiology. Through pathway analysis of the identified phosphorylation sites, we found Extracellular Signal-Regulated Kinase (ERK) signaling emerged as an important pathway; notably, upregulation of ERK1/2 phosphorylation was observed as a significant marker associated with AR. Importantly, targeting ERK inhibitors presents a potential therapeutic strategy for modulating key inflammatory response signaling pathways in the context of AR, although this finding is derived from preclinical mouse models and requires rigorous validation in human pediatric nasal mucosal tissues before any clinical translation can be considered. Collectively, these findings highlight that elucidating the molecular mechanisms underlying AR-induced nasal mucosal dysfunction in the mouse model may inform the novel therapeutic targets for pediatric allergy-related diseases. Overall, elucidating these mechanisms has substantial implications for developing targeted interventions aimed at mitigating inflammation associated with allergic rhinitis.

## 1 Introduction

Allergic rhinitis (AR) is a chronic, noninfectious inflammatory disorder of the nasal mucosa that is primarily mediated by immunoglobulin E (IgE), which is activated upon exposure to allergens (1). AR, with an increasing prevalence, is a significant global health challenge, currently affecting approximately 10–30% of adults and up to 40% of children worldwide (2, 3). This condition results in substantial economic burdens and adverse effects on overall health. It is recognized as the most common chronic condition among pediatric populations. The primary clinical manifestations of AR include rhinorrhea, nasal congestion, nasal itching, and sneezing (4). Although these symptoms may appear mild, their impact on children should not be underestimated. Approximately 20% of children exhibit AR symptoms between the ages of 2 and 3 years; this figure increases to approximately 40% by age six and remains at approximately 30% during adolescence (5). The increasing incidence and financial implications associated with AR underscore the urgent need for a deeper understanding of its molecular mechanisms to facilitate the development of targeted therapeutic interventions.

AR arises from a complex etiology involving both genetic and environmental components. Previous studies have shown that exposure to environmental agents—including allergens, air pollutants, climate variables, and viruses—is a major factor in the growing prevalence of AR (6, 7). Moreover, alterations in the levels of certain posttranslational modifications (PTMs) with a particular focus on phosphorylation may play a role in the development of AR. It has been reported that phosphorylation modulates immune responses by altering the activity of key proteins involved in inflammation and immune cell activation (8, 9). In the context of asthma and other respiratory diseases, dysregulated phosphorylation has been associated with aberrant immune responses and airway hyperreactivity. Specifically, published studies have demonstrated that phosphorylated p38 Mitogen-Activated Protein Kinase (MAPK) promotes the production of T helper 2 (Th2) cytokines, such as interleukin (IL)-4 and IL-5. This process is central to eosinophilic inflammation in asthma. Additionally, phosphorylated c-Jun N-terminal kinase (JNK) contributes to the apoptosis of airway epithelial cells, which compromises the integrity of the airway barrier. Additionally, phosphorylated Extracellular Signal-Regulated Kinase (ERK) plays a role in airway hyperreactivity and remodeling in chronic asthma (10).

Previously published studies have established that phosphorylation of kinases, including MAPK and Nuclear Factor-kappa B (NF-κB), play pivotal roles in the pathogenesis of airway inflammatory diseases. House dust mite-induced p38 MAPK phosphorylation promotes activator protein-1 activation, which in turn upregulates IL-24 to trigger signal transducer and activator of transcription 1/3 (STAT1/STAT3) phosphorylation, ultimately impairing nasal mucosal barrier function and promoting type 2 inflammation (11). In a similar manner, The IL-6/STAT3 signaling pathway, which phosphorylates NF-κB p65 to promote Th17/Th2 responses, has been implicated in the exacerbation of nasal inflammation and the progression of AR (12). The targeting of these kinase-driven pathways has demonstrated potential in the mitigation of allergic inflammation, as evidenced by studies in which kinase inhibitors were used to suppress cytokine release and immune cell infiltration (13–15). A comprehensive and detailed understanding of the phosphoproteomic landscape of AR has the potential to reveal novel biomarkers and therapeutic strategies that may alleviate symptoms and modify disease progression.

As a disorder mediated by allergens, AR involves the initiation of a specific immune response and inflammatory cascade in the nasal passages. However, whether AR alters the function of the nasal mucosa through phosphoproteomic mechanisms remains unclear. In this study, we established a model of AR in 3-week-old mice to simulate conditions in children prior to adulthood. We subsequently investigated the changes in global phosphorylation induced by AR in the nasal mucosa and explored potential functional abnormalities associated with these changes. Our proteomic analysis revealed that AR led to significant alterations in protein expression within the nasal mucosa, particularly affecting proteins known to be involved in immune responses. Through pathway analysis, the “ERK signaling pathway” emerged as an important pathway; notably, the upregulation of ERK phosphorylation was found to be a significant marker associated with AR-affected nasal mucosa. Importantly, our findings indicated that treatment with an ERK inhibitor conferred protection against ovalbumin (OVA)-induced AR. Mechanistically, ERK appears to regulate immune responses related to AR while alleviating inflammation. In summary, our study aimed to elucidate the epigenetic mechanisms underlying changes in the nasal mucosa induced by AR and explored potential therapeutic strategies for patients with AR complicated by immune responses related to nasal mucosal dysfunction.

## 2 Materials and methods

### 2.1 Patient cohort and ethics statement

This prospective study recruited pediatric participants from the Otolaryngology Head and Neck Surgery and Nutrition Departments of the Children’s Hospital affiliated to the Capital Institute of Pediatrics (Beijing, China) between October 2023 and December 2024. The study protocol was approved by the hospital’s Ethics Committee (Approval No. SHERLL2023088), and written informed consent was obtained from all participating children and their parents. Children in the AR group were recruited from the Department of Otolaryngology Head and Neck Surgery. A total of 125 pediatric patients with AR and 125 age-matched healthy control subjects were enrolled in this prospective clinical cohort. They met the following inclusion criteria: (1) age above 2 years; (2) diagnosis of AR according to Allergic Rhinitis and its Impact on Asthma (ARIA) guidelines, accompanied by relevant clinical symptoms; (3) a positive skin prick test result and elevated serum-specific IgE (sIgE > 0.35 KU/L, Phadia ImmunoCAP, Uppsala, Sweden) to at least one relevant allergen (16). Healthy control children were recruited from the Department of Nutrition and were confirmed by a physician to be free of allergic rhinitis or other allergic diseases. The exclusion criteria for all participants included: (1) history of nasal surgery; (2) presence of an upper respiratory infection or chronic rhinosinusitis at the time of enrollment; (3) diagnosis of any autoimmune or immunodeficiency disorder. The patients’ basic information was meticulously documented, including gender, age, family history of allergies, and other relevant details, while blood count data was collected.

### 2.2 Total nasal symptom score

The total nasal symptom score (TNSS) was employed to assess AR severity. This instrument rates three symptoms—rhinorrhea, sneezing/nasal itching, and nasal blockage—each on a 0–3 scale (0, none; 1, mild; 2, moderate; 3, severe). A total score was calculated by summation, with increasing values indicating worsening symptom burden (17).

### 2.3 Animals

The investigation utilized three-week-old male wild-type C57BL/6 mice. The reagents were procured from SPF (Beijing) Biotechnology Co., Ltd. The protocols for animal studies were approved by the Ethics Committee of Capital Institute (Beijing, China) (Ethic Certificate No: DWLL2023013). In each part, the experimental animals were grouped randomly to reduce deviation. The animal model of AR was constructed as previously described (18). In summary, 50 μg OVA (Sigma–Aldrich, St. Louis, MO, USA) and 1 mg aluminum hydroxide (Thermo Fisher, CA, USA) were dissolved in saline, after which the mixture was administered intraperitoneally to the mice on days 1, 7, and 14 (sensitization stage). The mice were subsequently subjected to intranasal instillation of 20 μL of a 10% OVA solution in each nostril on days 21 to 28. In the ERK inhibition group, PD98059 (MedChem Express, Monmouth Junction, NJ), a selective inhibitor of ERK1/2, was administered intranasally (19–21). The 10 mg/kg dose was administered to each nostril starting one hour after each OVA challenge from days 21 to 28 of the experimental protocol. All *in vivo* functional experiments (behavioral detection, HE and PAS histopathological staining) utilized six biological replicate mice per group (n = 6). For proteomic and phosphoproteomic LC-MS analysis, nasal mucosal tissues from 20 individual mice were pooled to form one single biological sample, and three independent batches of such pooled samples were prepared as biological replicates (n = 3 for CON group, n = 3 for AR group).

### 2.4 Behavioral tests

On the final experimental day (day 28), an intranasal OVA challenge was administered. Nasal symptoms were then immediately assessed by two blinded researchers during a 10-minute observation window, with a focus on counting episodes of sneezing and nasal rubbing (n = 6 per group).

### 2.5 Histopathological analyses of nasal tissues

A 10% neutral buffered formaldehyde solution was used to fix the heads of the mice (Sigma–Aldrich, St.) for one week. The heads of the mice were then decalcified in 0.1 M EDTA buffer (Biosolution, Suwon, Korea) and embedded in paraffin for two weeks. The tissue blocks were cut into 5 µm thick sections and some of them were stained with hematoxylin (Sigma–Aldrich, St.) and eosin (Sigma–Aldrich, St. Louis, MO, USA). The remaining sections were stained with periodic acid–Schiff (PAS; Sigma–Aldrich, St. Louis, MO, USA) to analyze goblet cell hyperplasia. A slide digital scanner from 3DHISTECH (Budapest, Hungary) was used to scan the slides, which were then read with Panoramic case software (3DHISTECH, Budapest, Hungary). Six nasal mucosa tissue samples from each group were used for the analyses.

### 2.6 Protein extraction and trypsin digestion

Protein extraction was initiated by cryogenically pulverizing tissue samples in liquid nitrogen. The resulting powder was transferred to a 5 mL centrifuge tube and combined with four volumes of lysis buffer (8 M urea, 1% protease inhibitor cocktail). The mixture was subjected to three cycles of sonication on ice using a high-intensity ultrasonic processor (Scientz). Subsequent centrifugation at 12,000 g (4 °C, 10 min) removed insoluble debris. The supernatant was collected, and protein concentration was quantified using a BCA assay kit per the manufacturer’s protocol. For protein purification, the sample was vortexed with one volume of pre-chilled acetone, followed by addition of four further volumes of acetone and precipitation at -20 °C for 2 h. The pellet was washed 2–3 times with pre-chilled acetone, then reconstituted in 200 mM TEAB and dispersed by sonication. Digestion was performed by adding trypsin at a 1:50 (w/w) enzyme-to-protein ratio for overnight incubation. Prior to digestion, proteins were reduced with 5 mM dithiothreitol (56 °C, 30 min) and alkylated with 11 mM iodoacetamide (room temperature, 15 min, in darkness). Finally, peptides were desalted using a Strata X SPE column.

### 2.7 Liquid chromatography–mass spectrometry (LC–MS) analysis

Following tryptic digestion, peptides were reconstituted in solvent A and injected onto a custom reversed-phase analytical column (100 μm i.d. × 25 cm). Separation was performed on a NanoElute UHPLC system (Bruker Daltonics) using a binary solvent system: solvent A (0.1% formic acid, 2% acetonitrile in water) and solvent B (0.1% formic acid in acetonitrile). A linear gradient was applied at a constant flow rate of 500 nL/min as follows: 6–24% B over 0–14 min, 24–35% B over 14–16 min, 35–80% B over 16–18 min, followed by 80% B from 18 to 20 min. Eluted peptides were ionized via a capillary source at an electrospray voltage of 1.75 kV and analyzed on a timsTOF Pro 2 mass spectrometer operated in data-independent acquisition parallel accumulation serial fragmentation (dia-PASEF) mode. Full MS scans were acquired across the m/z range 300–1500. MS/MS spectra were collected in dia-PASEF mode with an isolation window of 7 m/z, covering a scan range of 400–850 m/z, and 20 PASEF MS/MS scans were acquired per cycle. Raw data were processed using Spectronaut (v.18) with search against the Mus_musculus_10090_SP_20231220.fasta database (17,191 entries) concatenated with a reverse decoy. Search parameters included: enzyme, trypsin/P (max 2 missed cleavages); fixed modification, carbamidomethyl (C); variable modifications, acetylation (protein N-terminus) and oxidation (M). Identification false discovery rates (FDR) were set at < 1% at the protein, peptide, and PSM levels.

### 2.8 Motif analysis

Motif analysis was conducted on the MoMo platform based on the motif-x algorithm. Peptide sequences encompassing 6 amino acids upstream and downstream of each identified modification site were extracted. The background dataset consisted of analogous sequences from all potential modification sites. A characteristic sequence was considered a significant motif if it was represented in over 20 peptides and satisfied a statistical threshold of *p* < 0.000001.

### 2.9 Accession codes

The mass spectrometry proteomics data have been deposited to the ProteomeXchange Consortium (https://proteomecentral.proteomexchange.org) via the iProX partner repository with the dataset identifier PXD073099.

### 2.10 Statistical analysis

#### 2.10.1 Proteomic and phosphoproteomic differential analysis

Quantitative data were normalized using Spectronaut software. For two-group comparisons, statistical significance was assessed via two-tailed Student’s *t*-test. Differentially expressed proteins and phosphosites were identified using the following thresholds: *p* < 0.05 and absolute fold change ≥ 1.5 (i.e., ≥1.5 or ≤0.667). Functional enrichment analyses—including Gene Ontology (GO), Kyoto Encyclopedia of Genes and Genomes (KEGG), and WikiPathways—were performed using the two-tailed Fisher’s exact test.

#### 2.10.2 Cytokine detection statistical methodology

Nasal mucosa cytokine expression levels were normalized to an internal reference protein. Intergroup differences were evaluated using an unpaired (independent-samples) Student’s *t*-test. When multiple comparisons were conducted, appropriate correction (e.g., Bonferroni or Benjamini–Hochberg) was applied.

#### 2.10.3 Other endpoints (behavioral scores, histopathological scores, and clinical indicators)

Two-group comparisons were performed using Student’s *t*-test. All statistical analyses were carried out using GraphPad Prism 5 and R (version 4.4.2); significance thresholds were uniformly defined as *p* < 0.05 (*), *p* < 0.01 (**), and *p* < 0.001 (***)—and these conventions were explicitly and separately specified for each of the three analytical platforms.

## 3 Results

### 3.1 Clinical characteristic of pediatric patients with allergic rhinitis

**Figure 1:**
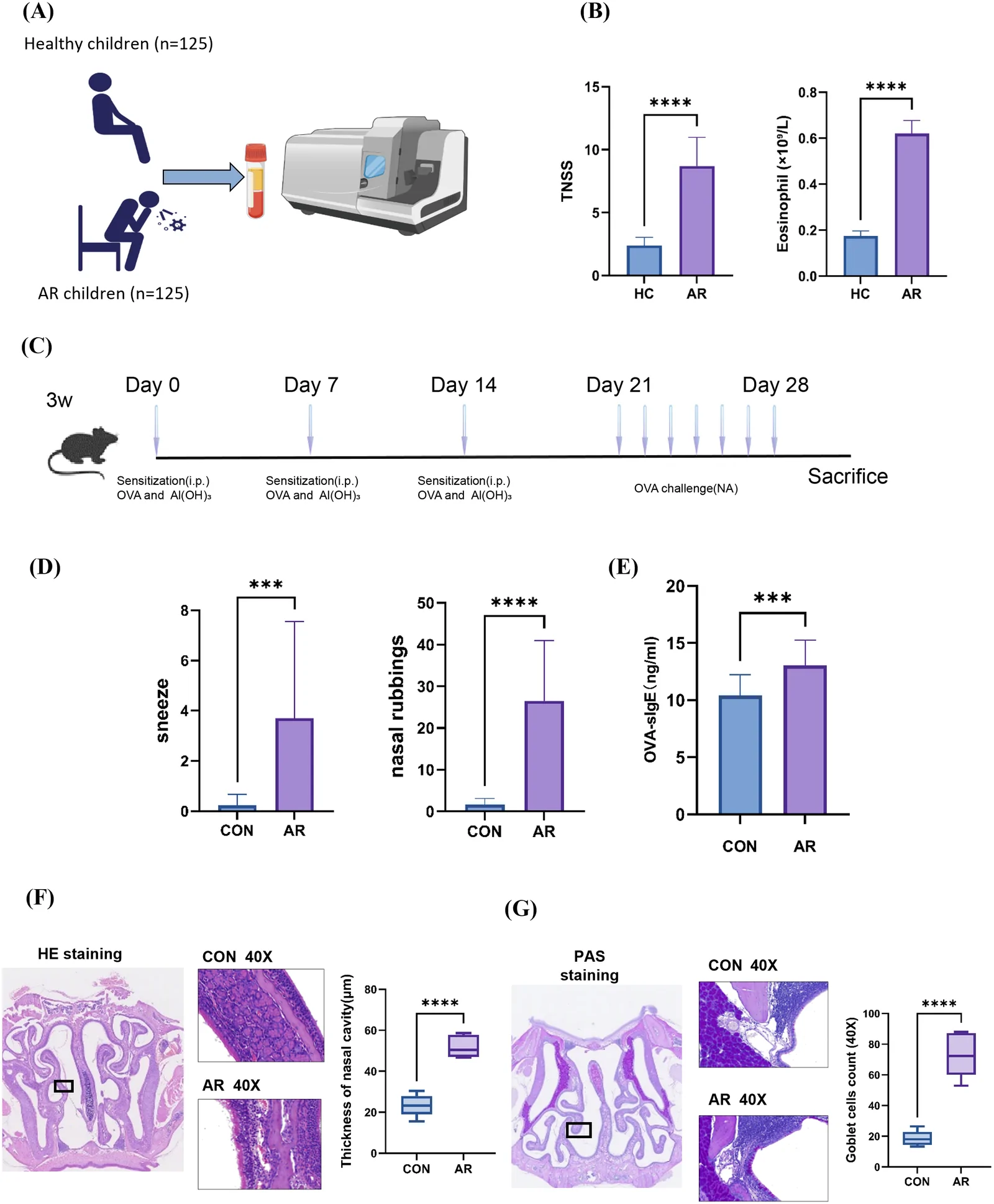
Clinical characteristics of the pediatric AR cohort and validation of the OVA-induced AR mouse model. **(A)** Flowchart illustrating the recruitment and grouping of pediatric participants: 125 children with allergic rhinitis (AR) and 125 healthy controls (HC). **(B)** Comparison of key clinical parameters between the AR and HC groups. Left: Total Nasal Symptom Score (TNSS, composite of rhinorrhea, sneezing/itching, and nasal blockage, each scored 0–3); right: peripheral blood eosinophil counts (×10^9^/L). Bars represent mean ± SD; *p < 0.001 by unpaired two-tailed Student’s t-test. **(C)** Experimental timeline for OVA-induced AR mouse model. Sensitization: intraperitoneal injection of OVA + aluminum hydroxide on days 1, 7, and 14. Challenge: intranasal instillation of 20 μL 10% OVA per nostril on days 21–28. Behavioral and tissue analyses were performed on day 28. **(D)** Nasal allergic behaviors quantified during a 10-min observation window immediately after the final OVA challenge. Left: sneezing frequency; right: nasal rubbing episodes (n = 6 per group). **(E)** Serum OVA-specific IgE (sIgE) levels measured by ELISA. AR group exhibited a marked increase versus CON (n = 6 per group). **(F)** Representative hematoxylin and eosin (HE)-stained nasal mucosal sections and corresponding quantification of mucosal thickness (n = 6 per group). **(G)** Representative periodic acid–Schiff (PAS)-stained sections and quantification of goblet cell hyperplasia (n = 6 per group). For panels (D–G)**, data are mean ± SEM; **p < 0.01, ***p < 0.001 by unpaired two-tailed Student’s t-test.

This study included 125 children with AR and 125 healthy control (HC) children (**Figure 1A**). There were no statistically significant differences between the two groups with respect to age (HC group: 7.0 ± 0.5 years; AR group: 6.9 ± 0.5 years; *P* = 0.104) or sex distribution (55 girls in each group). However, a significantly higher proportion of subjects with a family history of allergies was observed in the AR group than in the HC group (68% vs. 23%, *P* < 0.001). Furthermore, a statistically significant difference in the total nasal symptom score (TNSS) was detected between the two groups (8.7 ± 2.3 vs. 2.4 ± 0.5, *p* < 0.001). Additionally, a notable increase in peripheral blood eosinophil counts was detected (0.65 ± 0.21 ×10^9^/L vs. 0.18 ± 0.06 ×10^9^/L, *p* < 0.001). These findings suggest that patients with AR exhibit distinct differences from healthy controls with respect to both clinical symptoms and inflammatory markers (**Figure 1B**; **Table 1**).

**Table 1:** Comparison of clinical and laboratory characteristics between pediatric allergic rhinitis patients and healthy controls.

Characteristics	HC group	AR group	*P*-value
Cases	125	125	
Age, years (mean + SD)	7.0 ± 0.5	6.9 ± 0.5	0.104
Gender (n),			
Female	55	55	
Male	70	70	
Family history (n),			<0.001
Yes	23	68	
No	102	57	
TNSS (mean ± SD)	2.4 ± 0.5	8.7 ± 2.3	<0.001
Eosinophil (×10^9^/L, mean ± SD)	0.18 ± 0.06	0.65 ± 0.21	<0.001

### 3.2 Establishment and validation of the OVA-induced allergic rhinitis mouse model

Next, we established an AR model as previously described (**Figure 1C**). Compared with control (CON) mice, OVA-challenged AR mice exhibited significantly heightened allergic responses. Compared with the CON group, the OVA-challenged AR group demonstrated a marked increase in allergic reactions. Specifically, the number of sneezing episodes (mean ± standard error of the mean [SEM]: 3.71 ± 3.853 vs. 0.24 ± 0.437) and the frequency of nose-scratching behavior (mean ± SEM: 26.53 ± 14.418 vs. 1.65 ± 1.445) (**Figure 1D**) were significantly greater in AR mice (both *p* < 0.001), which are two hallmark clinical manifestations of allergic rhinitis observed in murine models. Concurrently, the serum level of sIgE against OVA—a crucial serological marker indicative of the activation of the allergic immune response—also substantially increased in the AR group compared with the CON group (**Figure 1E**, *p* < 0.01). The integration of these phenotypic and serological data collectively confirms the successful establishment of the OVA-induced AR mouse model. Histopathological examinations further corroborated the pathological changes characteristic of AR in the nasal mucosa. Hematoxylin and eosin (HE) staining revealed pronounced inflammation of the nasal mucosa in AR mice, which was not observed in the control (CON) group. The pathological features included epithelial defects within the nasal mucosa, extensive infiltration of eosinophils—hallmark inflammatory cells associated with type 2 allergic responses—and significant dilation and edema of blood vessels in the submucosal layer (**Figure 1F**). Periodic acid–Schiff (PAS) staining, a specific method for labeling mucin-secreting goblet cells, revealed additional pathological alterations in AR mice. The morphology of the goblet cells was irregular and was accompanied by marked hyperplasia, increased mucus secretion, and thickening of the nasal mucosal epithelium (**Figure 1G**). These histopathological findings were consistent with both the phenotypic and serological observations, thereby further validating the OVA-induced AR model for subsequent proteomic and phosphoproteomic analyses.

### 3.3 Identification of numerous proteins exhibiting differential expression in the proteome of the AR nasal mucosa

**Figure 2:**
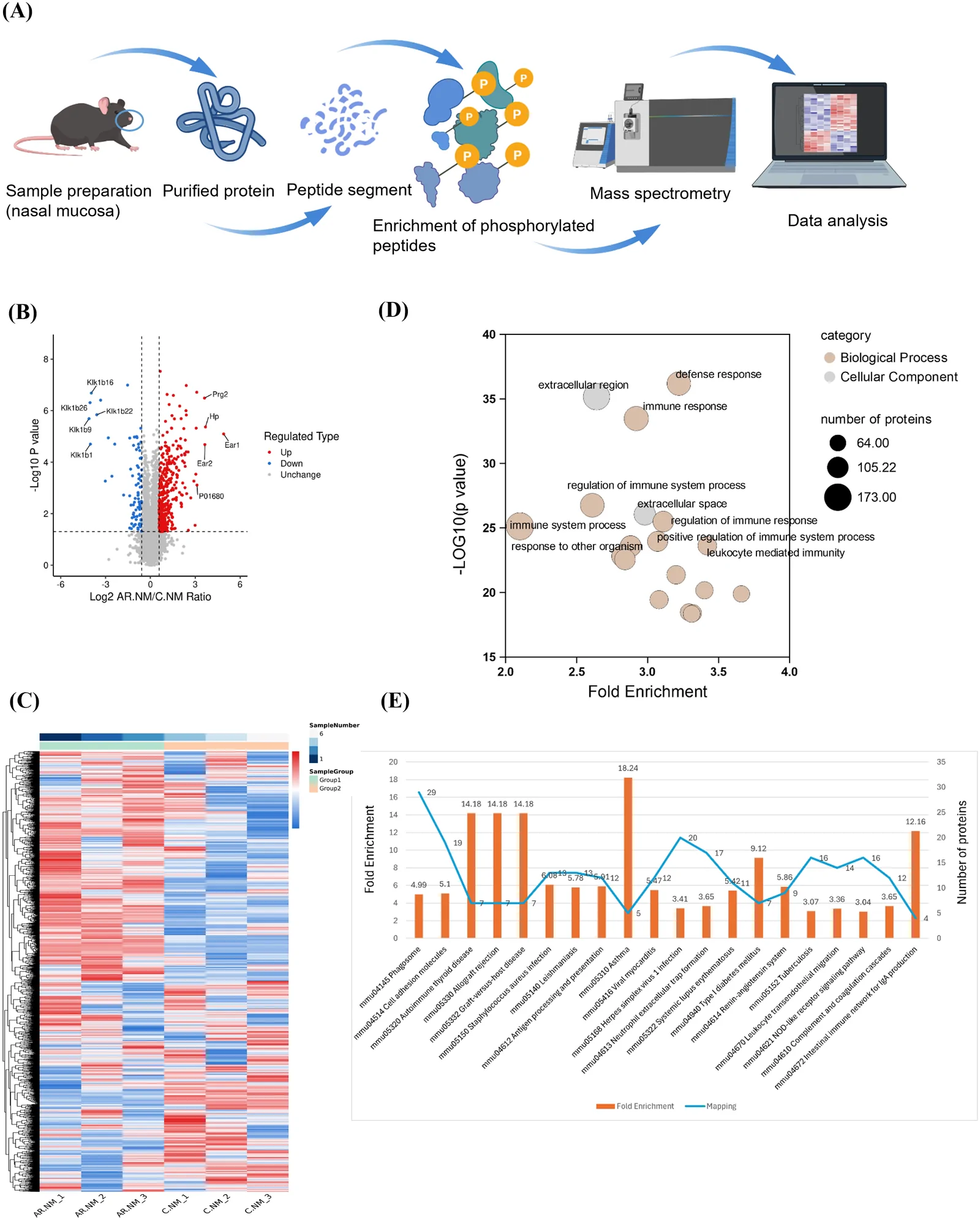
Proteomic profiling of differentially expressed proteins (DEPs) in the nasal mucosa of OVA-induced AR mice. **(A)** Workflow of label-free quantitative proteomic analysis: protein extraction, trypsin digestion, LC-MS/MS acquisition, and bioinformatic annotation. Each group contained 3 biological replicates (n = 3 per group, each pool from 20 mice). **(B)** Volcano plot of all quantified proteins. X-axis: log_2_ (fold change, AR vs CON); y-axis: −log_10_(*P*-value). Red dots: significantly upregulated proteins (fold change > 1.5, *P* < 0.05); blue dots: significantly downregulated proteins (fold change < 0.667, *P* < 0.05); gray dots: non-significant. Vertical dashed lines mark the fold-change thresholds; horizontal dashed line indicates *P* = 0.05. **(C)** Unsupervised hierarchical clustering heatmap of all DEPs. Each column represents an individual sample (AR1–3, CON1–3); each row represents a protein. Red: higher expression; blue: lower expression. **(D)** Bubble chart of the top 20 Gene Ontology (GO) biological process enrichment terms for DEPs. X-axis: fold enrichment; y-axis: enriched GO terms; bubble size: number of DEPs annotated to each term; bubble color: −log_10_ (adjusted *P*-value). **(E)** Combined bar-line chart of KEGG pathway enrichment for DEPs. Bars: number of DEPs annotated to each pathway (left y-axis); red line: −log_10_ (adjusted P-value, right y-axis). All enrichment analyses in **(D, E)** used two-sided Fisher’s exact test with Benjamini–Hochberg FDR correction (adjusted *P* < 0.05).

To assess the molecular impact of AR on the nasal mucosa, protein extractions were conducted from mouse nasal mucosal tissue, followed by LC–MS analysis (**Figure 2A**). This methodology was employed to analyze nasal mucosa samples from both the OVA-induced AR group and the control group. For proteomic and global phosphoproteomic profiling, nasal mucosa tissues of 20 mice were mixed as one biological replicate; three independent pooled sample batches were collected for the control group and AR group respectively, resulting in n = 3 biological replicates for mass spectrometry detection. The raw mass spectrometry data were processed using spectronaut software under stringent data filtration criteria (false discovery rate [FDR]<1% at both the peptide and protein levels) to ensure the reliability of the identification results. A total of 72,744 peptides were identified, including 69,925 unique peptides that could be distinctly mapped to specific proteins. These peptides corresponded to 8,529 proteins; among them, 8,498 proteins were successfully quantified, indicating extensive coverage of the nasal mucosa proteome and sufficient data depth for subsequent differential analysis (**Supplementary Figure S1A**). The Pearson correlation coefficient (PCC) was calculated on the basis of normalized protein quantification data, and a visual heatmap was generated to evaluate similarities between biological replicates as well as distinctions between groups (**Supplementary Figure S1B**). To identify proteins whose expression was significantly altered in the AR nasal mucosa, differentially expressed proteins (DEPs) were screened using predefined thresholds: a fold change (FC) > 1.5 for upregulation or a FC < 1/1.5 for downregulation and a *p* value < 0.05 determined by Student’s t test.

Applying these criteria, a total of 458 DEPs were identified between the AR and CON groups; specifically, among these DEPs, 376 upregulated proteins and 82 downregulated proteins were detected in the AR group compared with the CON group (**Figure 2B**; **Supplementary Table 1**). Heatmap analysis demonstrated that AR induced substantial alterations in the overall proteomic profile of the nasal mucosa (**Figure 2C**). This clustering analysis further validated the robustness of the differentially expressed protein (DEP) screening and revealed distinct proteomic signatures between the AR and control nasal mucosa.

To elucidate the biological roles of the 458 identified DEPs and their potential involvement in the pathogenesis of OVA-induced AR, functional annotation and enrichment analyses were conducted utilizing the Gene Ontology (GO) and Kyoto Encyclopedia of Genes and Genomes (KEGG) databases. To ensure the reliability of these biological interpretations, all enrichment results were filtered using a significance threshold of *p* < 0.05. The GO annotation system classifies gene/protein functions into three independent categories: biological process (BP), cellular component (CC), and molecular function (MF). In this study, significant enrichment was observed within several processes under the BP category, including defense response, immune response, regulation of immune system process and leukocyte-mediated immunity. The top 20 GO enrichment functions of the DEPs were plotted on the basis of fold enrichment, highlighting the top 10 entries. The CC categories included extracellular region and extracellular space (**Figure 2D**; **Supplementary Tables 2**, **3**). Within the MF category, TAP binding, peptidoglycan muralytic activity, immunoglobulin receptor activity, peptide antigen binding, and immunoglobulin receptor binding were the most enriched terms (**Supplementary Figure S1C**; **Supplementary Table 4**).

Next, a KEGG pathway enrichment analysis was performed to identify the signaling and metabolic pathways that are dysregulated in the nasal mucosa of individuals with AR. The most significantly enriched pathways included asthma and autoimmune thyroid disease, as indicated by their high fold enrichment values. Moreover, pathways such as phagosomes encompassed the greatest number of identified proteins, suggesting their broad functional relevance within the dataset (**Figure 2E**; **Supplementary Table 5**). Collectively, the results of these GO and KEGG enrichment analyses indicate that DEPs in the AR nasal mucosa are involved primarily in immune–inflammatory signaling pathways but also in metabolic processes to a lesser extent. These findings provide a comprehensive functional framework for elucidating the molecular mechanisms underlying ovalbumin-induced AR and highlight potential therapeutic targets.

### 3.4 Global phosphoproteomics of the nasal mucosa in AR mice

**Figure 3:**
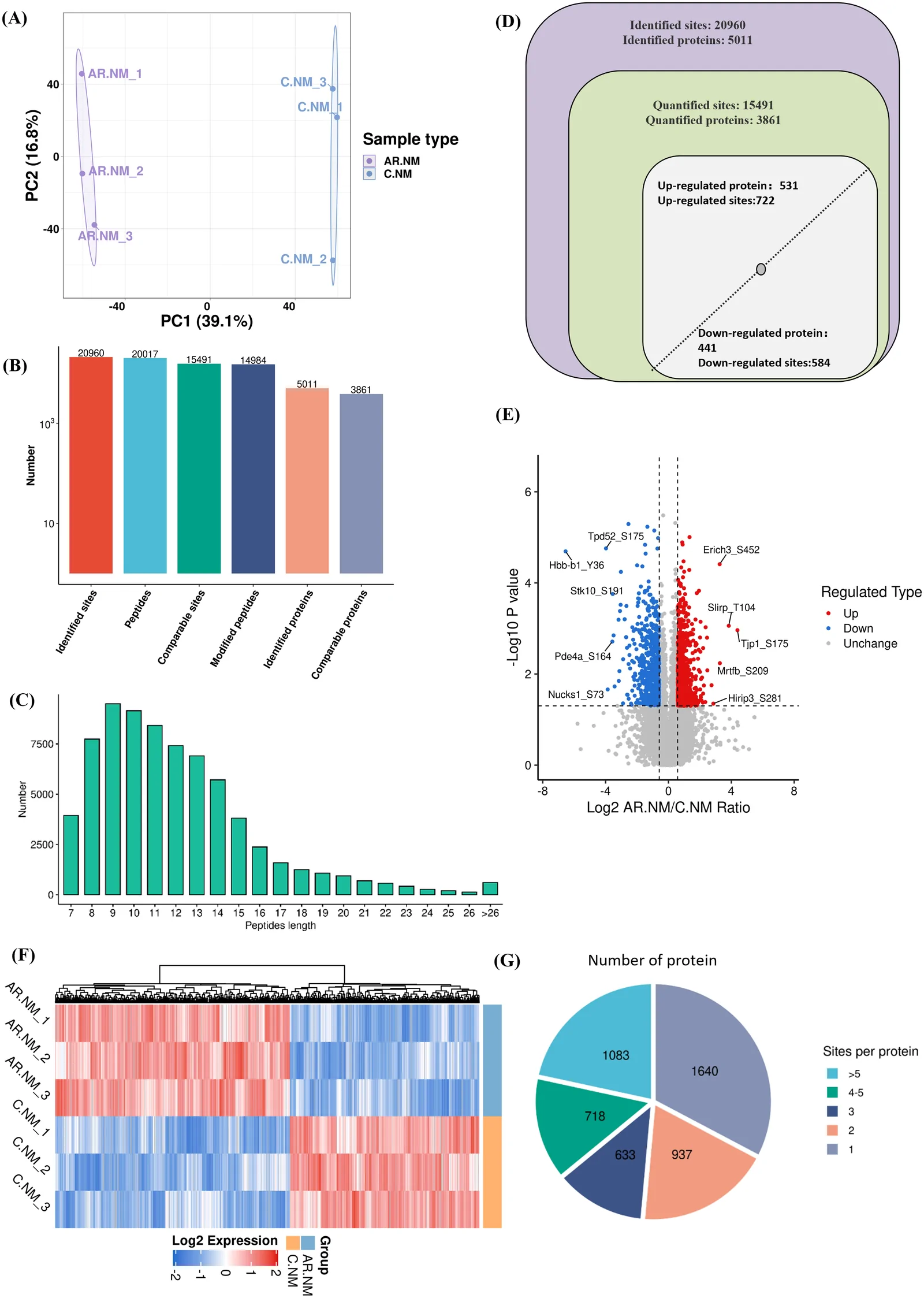
Global phosphoproteomic landscape of the nasal mucosa in AR mice. **(A)** Principal component analysis (PCA) plot of all quantified phosphosites. Each dot represents a biological replicate (n = 3 per group). **(B)** Summary bar chart of identified phosphorylation events: total spectra, modified peptides, unique phosphopeptides, and phosphosites identified after stringent FDR filtering (< 1% at both peptide and site levels). **(C)** Histogram showing the length distribution of identified phosphorylated peptides. **(D)** Pie chart summarizing the coverage of quantified phosphoproteins and phosphorylation sites. **(E)** Volcano plot of differentially phosphorylated sites. X-axis: log_2_ (fold change, AR vs. CON); y-axis: −log_10_ (*P*-value). Red: upregulated sites (fold change > 1.5, *P* < 0.05); blue: downregulated sites (fold change < 0.667, *P* < 0.05); gray: non-significant. **(F)** Unsupervised hierarchical clustering heatmap of all differentially phosphorylated sites. Columns: individual samples (AR1–3, CON1–3); rows: phosphosites. Red: higher phosphorylation; blue: lower phosphorylation. **(G)** Pie chart showing the distribution of phosphorylation sites per protein. Statistical significance in **(E)** was determined by unpaired two-tailed Student’s t-test with Benjamini–Hochberg FDR correction (FDR < 1%).

To obtain a comprehensive overview of the AR-regulated phosphoproteome, global phosphoproteomic analysis was conducted on the nasal mucosa from both the control (n = 3) and AR (n = 3) groups. High-performance liquid chromatography coupled with mass spectrometry (HPLC–MS) was employed to investigate phosphorylated peptides in the nasal mucosa of individuals with AR. A significance threshold of *p* < 0.05, along with a fold change greater than 1.5 or less than 0.667, was applied to refine the list of potentially relevant alterations in phosphorylation levels. Principal component analysis (PCA) demonstrated that samples from each group could be distinctly differentiated on the basis of their proximity to the AR group along principal components PC1 and PC2; conversely, samples within the control group exhibited similar distances among themselves (**Figure 3A**). Following stringent data filtration procedures, a total of 14,984 phosphorylation sites were successfully identified alongside 20,017 peptides and their corresponding modified forms (**Figure 3B**).

To ensure the reliability of these identification results, a quality control analysis assessing peptide length distribution was performed. This analysis revealed that peptide lengths ranged from 7 to 20 amino acids, which is consistent with established norms for enzymatic digestion and mass spectrometry fragmentation processes (**Figure 3C**). In total, the quantification efforts yielded data for 3,861 proteins encompassing an aggregate of 15,491 phosphorylation sites (**Figure 3D**). Interestingly, a total of 441 downregulated phosphorylation sites on 584 proteins and 531 upregulated sites on 722 proteins were identified in the nasal mucosa of the AR group (fold change > 1.5 or <0.667; *p* < 0.05) compared with those in the CON group (**Figure 3E**; **Supplementary Table 6**). The heatmap illustrates that the expression patterns of differentially modified sites across various replicate samples within the same group are highly similar; however, significant differences exist in the expression patterns of these modified sites between the AR and Con groups (**Figure 3F**). Among the phosphorylated proteins analyzed, 1640 exhibited a single modification site, while 1083 displayed more than five modification sites (**Figure 3G**; **Supplementary Table 7**). Additionally, specific proteins exhibited the highest number of modification sites, including Srrm2 (164 sites), Map1b (120 sites), Ank2 (107 sites), Srrm1 (67 sites), and Map1a (65 sites). These findings further reinforce the substantial disparities observed in phosphorylation levels between these two distinct sample groups.

### 3.5 Subcellular distribution and GO analysis of significantly up- and downregulation of phosphorylation of proteins

**Figure 4:**
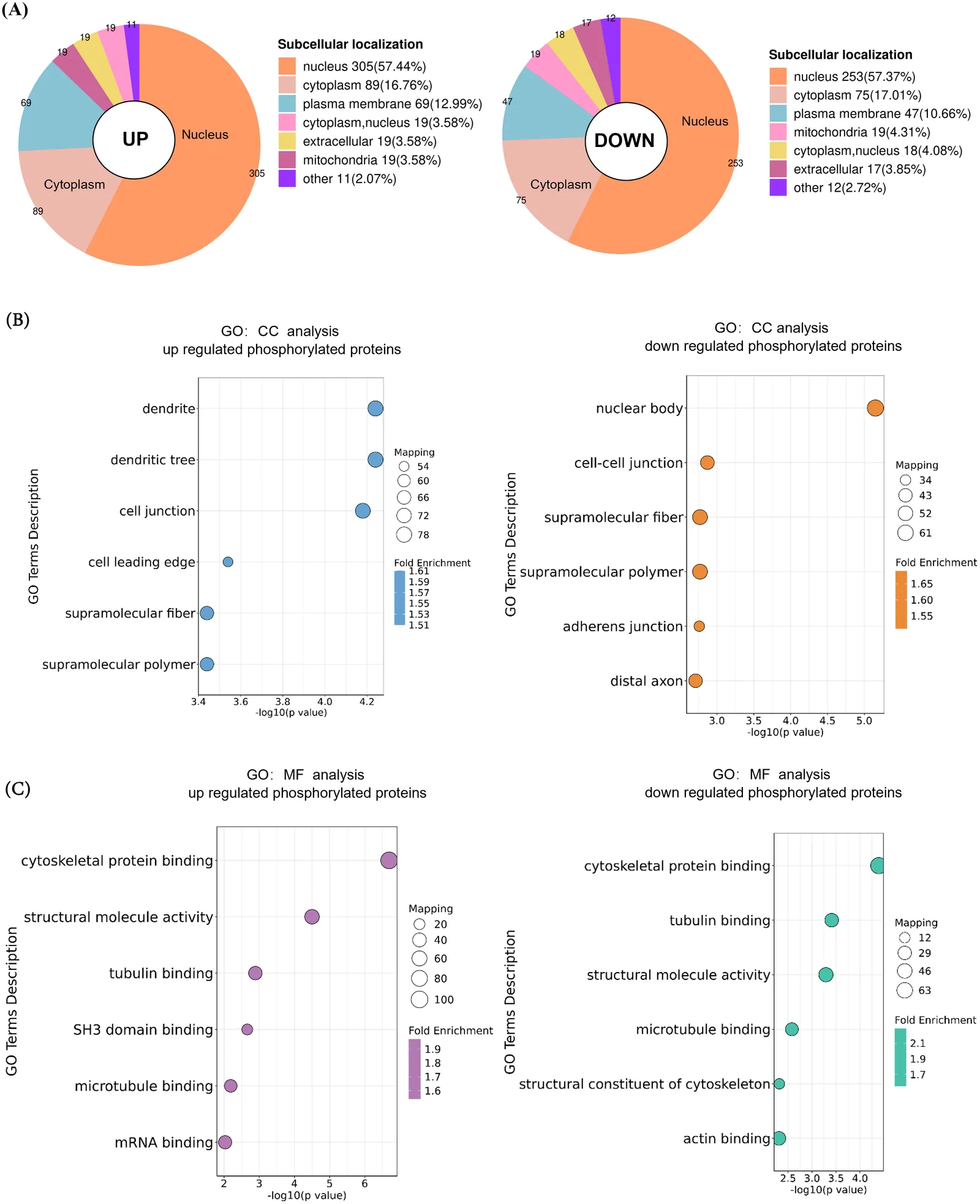
Subcellular localization and Gene Ontology (GO) enrichment (cellular component and molecular function) of differentially phosphorylated proteins. **(A)** Subcellular distribution of upregulated and downregulated phosphorylated proteins. Numbers above subcellular localizations indicate protein counts in each compartment. **(B)** Bubble chart of cellular component (CC) GO enrichment for differentially phosphorylated proteins. **(C)** Bubble chart of molecular function (MF) GO enrichment. All enrichment analyses used two-sided Fisher’s exact test with Benjamini–Hochberg FDR correction (adjusted *P* < 0.05).

There were notable differences in the subcellular localization of proteins whose phosphorylation was upregulated and downregulated. As illustrated in **Figure 4A**, the classification results indicated that these proteins were predominantly localized within the nucleus, cytoplasm, plasma membrane, extracellular space, mitochondria, and other cellular compartments. In the nucleus, a total of 305 modified proteins were upregulated, whereas 253 were downregulated. Within the cytoplasm, 89 upregulated and 75 downregulated modified proteins were identified. The plasma membrane contained 69 upregulated and 47 downregulated modified proteins. The distribution of the mitochondria was equal, with 19 modified proteins each for up- and downregulation. Additionally, in the extracellular space, 19 upregulated and 17 downregulated modified proteins were detected (**Supplementary Table 8**).

GO analysis annotation pertaining to CC revealed that differentially phosphorylated proteins were significantly enriched in dendrites compared with those whose expression was upregulated and in nuclear bodies compared with those whose expression was downregulated (**Figure 4B**; **Supplementary Table 9**). Furthermore, MF analysis demonstrated that both groups of differentially phosphorylated proteins were significantly enriched in protein folding and binding to cytoskeletal proteins (**Figure 4C**; **Supplementary Table 10**). These findings suggest that androgen receptor-induced changes in phosphorylation are primarily involved in distinct molecular functions associated with processing within cellular compartments such as the cytoplasm and nucleus. These findings suggest that under allergic rhinitis conditions, phosphorylation of proteins in various subcellular structures of the mouse nasal mucosa result in specific changes related to abnormal functional regulation within those structures. These findings provide valuable insights at the subcellular level for understanding the pathogenesis of allergic rhinitis.

### 3.6 Enrichment clustering analysis of biological processes and KEGG pathways in the phosphoproteomics of nasal mucosa from AR mice

**Figure 5:**
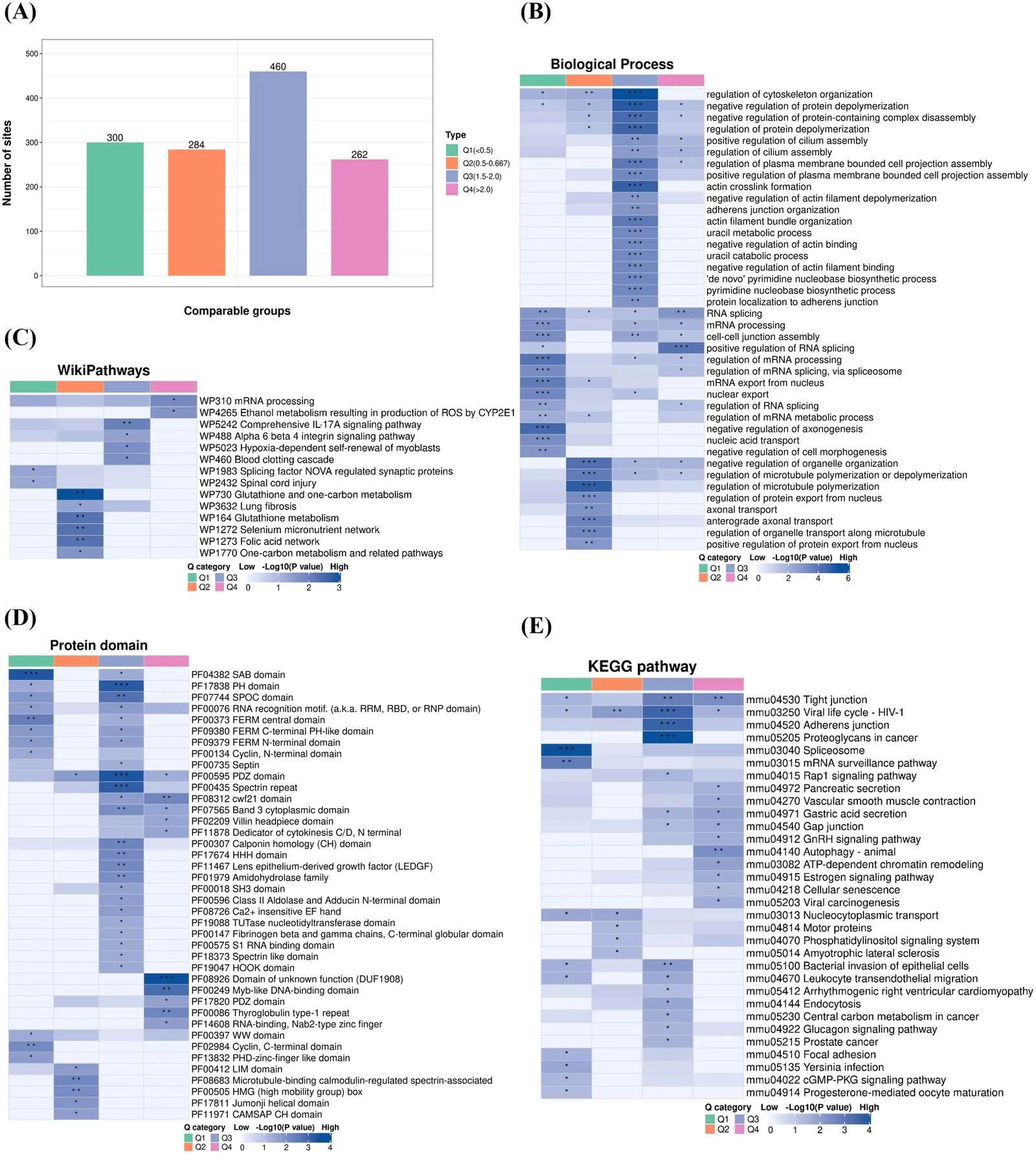
Functional stratification and enrichment analysis of the phosphoproteome in AR nasal mucosa. **(A)** Quartile distribution (Q1–Q4) of all differentially phosphorylated proteins ranked by fold change (AR vs. CON). Q1: most upregulated sites (highest fold change); Q4: most downregulated sites (lowest fold change); Q2 and Q3 represent intermediate changes. This grouping is consistently applied across panels **(B–E)** to reveal functional themes associated with different magnitudes of phosphorylation alteration. **(B)** Bar charts of Gene Ontology biological process (BP) enrichment in each quartile. **(C)** WikiPathways enrichment by quartile. **(D)** Protein domain enrichment by quartile. **(E)** KEGG pathway enrichment by quartile. All analyses in **(B–E)** used two-sided Fisher’s exact test with Benjamini–Hochberg FDR correction (adjusted *P* < 0.05).

To facilitate a comparative analysis of the functional disparities among proteins exhibiting differential alterations, the proteins were categorized into four distinct groups (Q1–Q4) on the basis of their differential expression fold changes (**Figure 5A**). Each group was subsequently subjected to a series of analytical procedures. Stratified GO biological process analysis revealed that both Q1 and Q4 were enriched in RNA-related processes, such as mRNA splicing and nuclear export. In contrast, Q2 was associated with microtubule polymerization and organelle organization, whereas Q3 was linked to cytoskeleton reorganization and plasma membrane projections (**Figure 5B**; **Supplementary Table 11**). Furthermore, WikiPathways analysis indicated that Q1 encompassed neuronal pathways, Q2 was related to glutathione metabolism, Q3 uniquely featured IL-17A signaling, and Q4 included pathways involved in mRNA processing and oxidative stress (**Figure 5C**; **Supplementary Table 12**). Protein domain analysis revealed that Q1 was characterized by SAB, FERM central, and C-terminal domains; Q2 contained microtubule-binding spectrin-associated domains along with HMG-box domains; and Q3 was enriched in PH, SPOC, and PDZ domains and spectrin repeats, whereas Q4 contained Cwf21, Myb-like DNA-binding domains, and thyroglobulin type-1 repeat domains (**Figure 5D**; **Supplementary Table 13**). Consistent with these findings, KEGG pathway analysis indicated that Q1 was enriched in genes related to spliceosome activity and mRNA surveillance mechanisms; Q2 was enriched in genes related to the HIV-1 viral life cycle and nucleocytoplasmic transport processes; and Q3 highlighted adherens junctions along with proteoglycans involved in cancer progression, whereas Q4 focused on genes related to tight junctions and autophagy pathways (**Figure 5E**; **Supplementary Table 14**). Collectively, these results illustrate a coherent functional stratification across the phosphoproteome—spanning from RNA processing and cytoskeletal remodeling to inflammatory signaling and metabolic regulation.

### 3.7 Role of ERK in the nasal mucosa of AR mice

**Figure 6:**
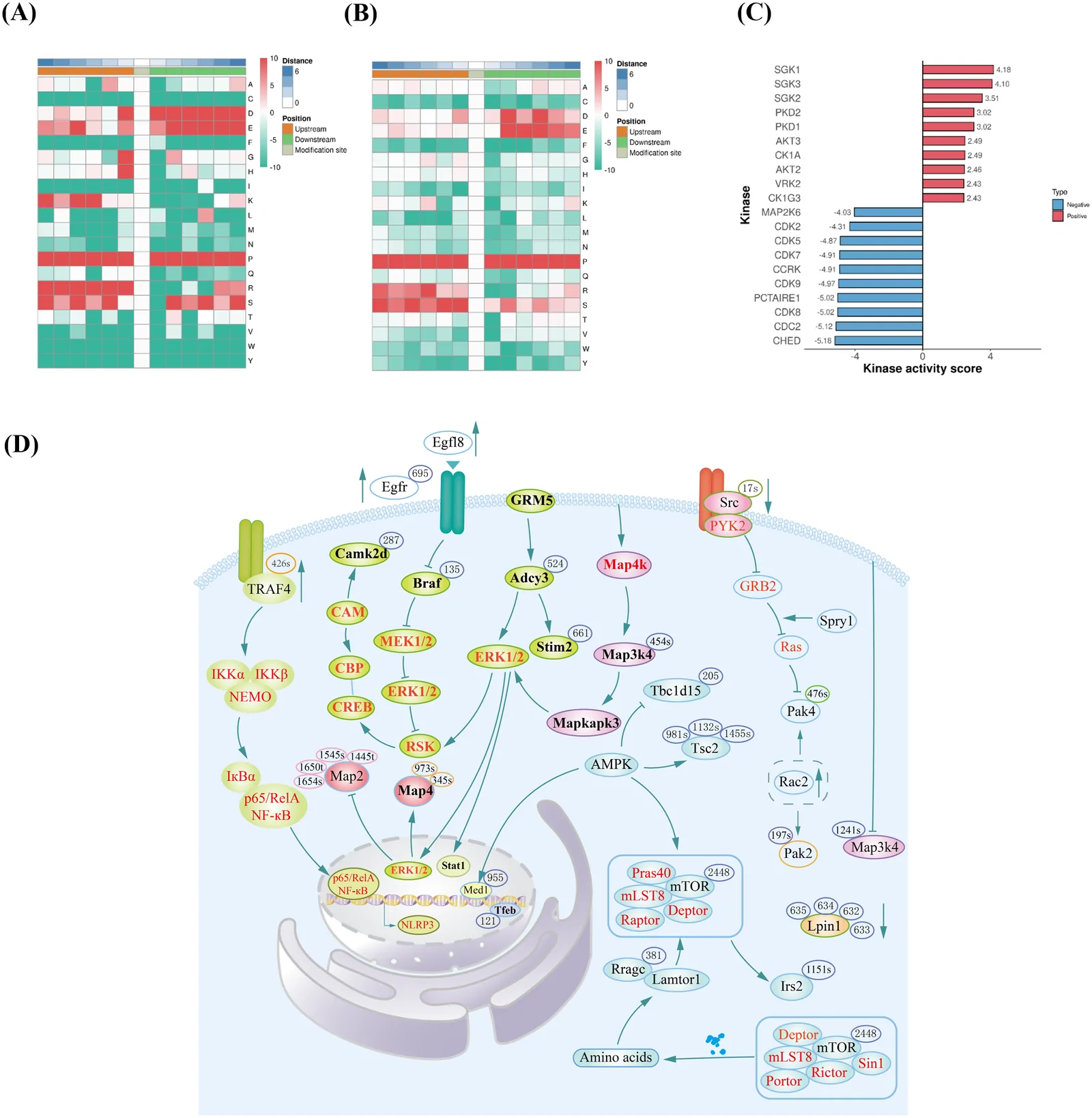
Kinase activity prediction and ERK-centered signaling network in AR nasal mucosa. **(A)** Consensus sequence motif of serine (Ser) phosphorylation sites. The motif logo displays amino acid preferences at positions −6 to +6 around the central phosphorylated Ser (position 0). **(B)** Consensus sequence motif of threonine (Thr) phosphorylation sites, presented in the same format as panel **(A)**. **(C)** Kinase activity scores derived from substrate motif enrichment analysis. Bars show the top 10 significantly activated kinases (red) and top 10 inhibited kinases (blue). Activity scores were calculated using two-tailed Student’s t-test (*P* < 0.05). **(D)** Integrated phosphorylation-dependent signaling regulatory network constructed from differentially phosphorylated proteins screened via nasal mucosal phosphoproteomics of OVA-induced AR mice; all omics data were derived from 3 pooled biological replicates, with each pool containing nasal mucosal tissues from 20 individual mice (n = 3). Oval nodes stand for proteins identified in this dataset; green ovals with bold text labels mark core upstream and downstream components of the ERK/MAPK cascade. Digits next to each protein label correspond to significantly changed phosphorylation residues detected by mass spectrometry. Solid directional arrows denote phosphorylation-mediated activation between upstream kinases and downstream substrates, while terminal lines without arrowheads represent inhibitory intermolecular interactions. Blue rectangular frames denote mTOR-related multi-protein functional complexes. Kinase activity analysis was performed using two-tailed Student’s t-test (*P* < 0.05).

To investigate the substrate preferences of enzymes involved in phosphorylation and to explore potential kinase–substrate interactions within the nasal mucosa of mice with AR, motif analysis was conducted on identified phosphorylation sites (serine and threonine) utilizing the MoMo tool based on the motif-x algorithm (**Figures 6A, B**). This analysis established a foundation for identifying key regulatory kinases involved in AR pathology (**Supplementary Table 15**). For these phosphorylation sites, we predicted their upstream phosphokinase relationships on the basis of sequence similarity. Initial predictions revealed a total of 50,875 regulatory relationships among 343 protein kinases and 4,894 phosphorylation sites across 1,622 proteins. We performed predictive analyses of phosphokinase activity by comparing the two groups. The results indicated enrichment in phosphokinase activity, with a P value less than 0.05; specifically, 47 kinases tended to be inhibited, while 19 were activated. A bar chart was used to illustrate the top ten phosphokinases with the highest activity scores under significantly activated or inhibited states (**Figure 6C**; **Supplementary Table 16**). A comprehensive enrichment analysis of differentially phosphorylated proteins was performed, resulting in the identification of several significantly enriched signaling pathways. To visually illustrate the interactions among these proteins within complex regulatory networks, an integrated signaling pathway diagram was constructed. Notably, this diagram includes key pathways such as the MAPK/ERK and NF-κB signaling cascades. It delineates a meticulously calibrated regulatory network in the allergic rhinitis model that spans from growth factor/inflammatory signal receptors to ERK activation, ultimately governing intracellular transcriptional processes. This encompasses various phosphorylation sites on mitogen-activated protein (MAP) 2, including S1545, S1654, T1445, and T1650, as well as phosphorylation sites on MAP4, such as S973 and S345 (**Figure 6D**). It is important to highlight that numerous differentially phosphorylated proteins—such as mechanistic target of rapamycin (mTOR) and AMP-activated protein kinase (AMPK)—are positioned at critical nodes within signal transduction pathways. This suggests that these molecules may serve as essential targets for the phosphorylation-mediated regulation of inflammatory responses associated with AR. Our findings indicate that disordered phosphorylation dynamics related to ERK signaling may substantially contribute to the impaired mucosal immune responses observed in the nasal mucosa of AR mice.

### 3.8 Pharmacological inhibition of ERK in the nasal mucosa relieves AR allergy symptoms and decreases the inflammatory response

**Figure 7:**
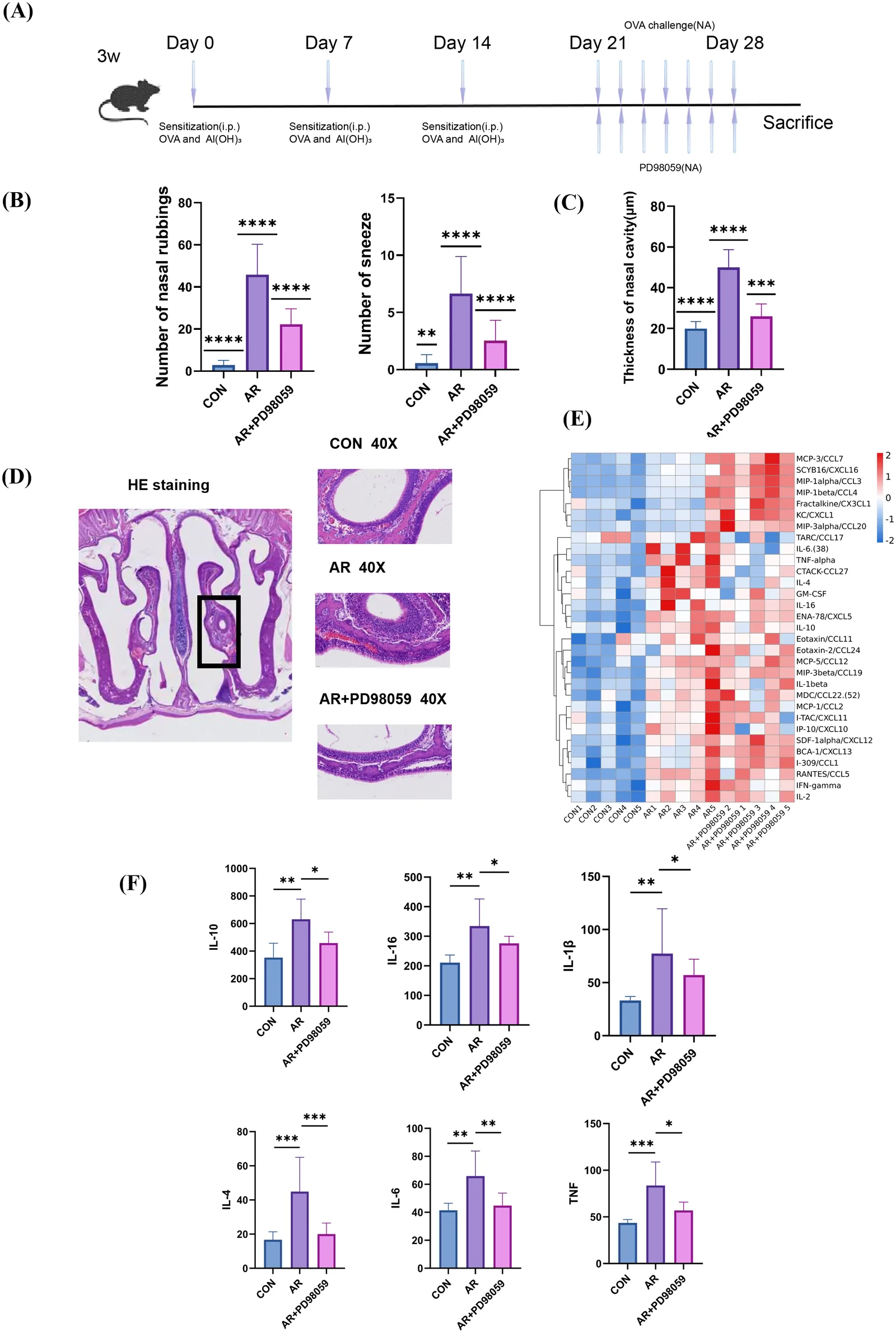
Therapeutic effects of ERK inhibition on OVA-induced AR in mice. **(A)** Schematic diagram of the experimental workflow for ERK inhibitor (PD98059) intervention. Mice were sensitized and challenged with OVA as in **Figure 1C**. From days 21–28, the ERK inhibitor group (AR+PD98059) received intranasal PD98059 (10 mg/kg per nostril, 1 h after each OVA challenge). All analyses were performed on day 28. **(B)** Nasal allergic behaviors (sneezing and rubbing) quantified during a 10-min observation window after the final challenge (n = 6 per group). **(C)** Quantification of nasal mucosal thickness from HE-stained sections (n = 6 per group). **(D)** Representative HE-stained nasal mucosal sections from CON, AR, and AR+PD98059 groups. **(E)** Heatmap of 31 cytokines measured in nasal mucosal homogenates by multiplex Luminex assay. Each column represents an individual mouse; each row represents a cytokine. **(F)** Relative expression levels of selected key inflammatory cytokines across the three groups. Bars represent mean ± SEM (n = 6 per group). Statistical comparisons in panels **(B–F)** were performed using one-way ANOVA followed by Tukey’s multiple comparisons test. *P < 0.05, **P < 0.01, ***P < 0.001; ns, not significant.

To further investigate the role of ERK in AR, we modulated ERK activity in the nasal mucosa of mice using the ERK inhibitor PD98059 administered via nasal drops daily for 7 days (**Figure 7A**). We then assessed the effects of ERK inhibition on AR-related symptoms and pathological changes. We observed that compared with AR group, PD98059 treatment led to a noticeable reduction in nasal rubbing and sneezing behaviors (**Figure 7B**). Furthermore, histological examination revealed a decrease in nasal cavity width and a reduction in the number of goblet cells in the nasal submucosa in the PD98059-treated group compared with those in the AR control group (**Figures 7C, D**). These results suggest that inhibition of ERK signaling in the nasal mucosa alleviates AR symptoms and associated tissue remodeling. We next measured the expression of 31 cytokines in the nasal mucosa following 7 days of intranasal administration of the ERK inhibitor PD98059. The results revealed that the expression levels of a number of cytokines, especially cytokines related to inflammation and immune responses, such as IL-10, IL-4, IL-6, and tumor necrosis factor (TNF), were altered after PD98059 treatment (**Figures 7E, F**). After standard curve fitting, data quality control, and normalization as described in the statistical methods, the expression levels of multiple inflammatory and immune-related cytokines were analyzed. These findings suggest that ERK signaling may play a regulatory role in the inflammatory response of the nasal mucosa in AR.

## 4 Discussion

Allergic rhinitis is a highly prevalent chronic inflammatory disorder that not only severely disrupts children’s sleep quality and daily activities through symptoms such as nasal itching, sneezing, and nasal congestion but also frequently accompanies complications such as asthma and sinusitis, imposing long-term adverse effects on the physical and mental health as well as growth and development of affected children (22, 23). Current research has clarified its core pathological features mediated by IgE and driven by gene–environment interactions, with symptomatic treatments available clinically that are, however, mostly confined to symptom alleviation (24, 25). Nevertheless, the molecular mechanisms by which posttranslational modifications, particularly phosphorylation, regulate nasal mucosal immune imbalance and functional disorders remain incompletely elucidated, leading to a lack of precise targeted curative strategies—a critical gap awaiting breakthrough in current studies (26, 27). In this study, we first delineated the clinical phenotypic characteristics in a pediatric AR cohort, including elevated allergic symptoms and eosinophil infiltration. To understand the molecular mechanisms underlying nasal mucosal dysfunction in AR, we employed a mouse model that recapitulates key features of pediatric AR and performed integrated proteomic and phosphoproteomic analyses. Specifically, we applied an integrated proteomic and phosphoproteomic approach to characterize the molecular landscape of the nasal mucosa in a murine model of OVA-induced allergic rhinitis (28, 29). Our analyses revealed extensive dysregulation at both the protein abundance and phosphorylation level, offering novel insights into the posttranslational mechanisms underlying AR pathogenesis. Specifically, our phosphoproteomic data showed that ERK signaling emerged as a key regulatory kinase pathway participating in nasal mucosal inflammation in mice—a finding that may partially account for the inflammatory pathogenesis underlying the clinical phenotypes in children with AR. However, our phosphoproteomic data also concurrently enriched other kinase pathways, including p38 MAPK, JNK, and NF-κB, indicating that ERK serves as an important convergence point rather than the sole primary upstream initiator of allergic inflammation. These results provide potential therapeutic targets for childhood AR, meriting further validation in human nasal mucosal samples.

Our study performed a systematic analysis of nasal mucosa tissue utilizing high-throughput proteomics technology, successfully identifying a substantial number of protein expression profiles and identifying 458 DEPs, comprising 376 upregulated proteins and 82 downregulated proteins. Our functional enrichment analysis of these DEPs revealed the molecular dysfunction characteristics of the nasal mucosa in the pathological context of AR (30). The upregulated DEPs are predominantly enriched in biological processes associated with immune activation, including type I interferon-mediated signaling pathways and negative regulation of lymphocyte-mediated immunity (31–33). Our data showed that their cellular localization is primarily concentrated within subcellular structures such as secretory granules and the extracellular matrix (34, 35). This finding indicates that active synthesis and secretion of immune-active molecules in the nasal mucosa are affected by AR, which is closely linked to the pathological features characterized by local inflammatory infiltration and hypersecretion of mucus. It is important to highlight that the pattern of immune activation observed here significantly differs from the type 2 inflammatory characteristics seen in CRSwNP (36–38). Recent studies have indicated that the expression levels of proteins such as glial cell line-derived neurotrophic factor (GDNF) and monocyte chemoattractant protein-4 (MCP-4) in the nasal fluid of patients with CRSwNP are correlated with disease severity (39, 40). By contrast, published data indicate that AR predominantly features the activation of type I interferon pathways (41, 42). This distinction may reflect an imbalance between Th1 and Th2 cytokines across these different conditions (43, 44). In contrast, the downregulated DEPs are significantly enriched in pathways related to metabolic regulation and vascular function, such as the tissue kinin release-kinin cascade and the renin-angiotensin system (45–47).

This study not only addresses a gap in existing research that predominantly emphasizes immune mechanisms while overlooking metabolism-related pathways but also offers a novel perspective for understanding the pathophysiological mechanisms underlying AR. Notably, the enrichment characteristics of immune inflammatory pathways identified in the proteomics data—such as asthma-related pathways and type I interferon signaling—align with the traditional view of AR as a condition primarily driven by type 2 inflammation (48–51). However, our phosphorylated proteomics data provide a more nuanced regulatory perspective, indicating that the phosphorylation-mediated regulatory network encompasses not only classical immune pathways but also fundamental cellular processes, including cytoskeletal reorganization, RNA processing, and cell junction integrity (52–55). This indicated that AR may modulate cellular responses to metabolic stress via phosphorylation-mediated modifications. Recent studies have shown that the overproduction of reactive oxygen species (ROS) and mitochondrial dysfunction contribute significantly to the activation of NF-κB, which ultimately results in inflammation of the nasal mucosal epithelium (56–58). Therefore, our study provides new insights into the potential mechanisms underlying inflammatory responses in AR. The extensive regulatory role of inflammatory signaling pathway within the phosphorylation network is closely linked to the dual imbalance in immune activation.

Our findings indicate that AR significantly alters phosphorylation-mediated cytoskeletal remodeling and the regulation of RNA metabolism, which are critical mechanisms in the pathophysiology of AR (59, 60). Collectively, these changes contribute to epithelial barrier dysfunction, immune cell infiltration, and the reprogramming of inflammatory gene expression (61). Stratified phosphoproteomic analysis (Q1–Q4) revealed significant functional partitioning on the basis of the magnitude of phosphorylation changes. The coordinated enrichment of cytoskeleton-related processes in moderately altered phosphoproteins (Q2/Q3), along with the prominence of cell junction pathways identified through KEGG analysis, suggests that phosphorylation-mediated cytoskeletal remodeling serves as a central mechanism in AR pathophysiology (62–64). Among the phosphorylated proteins that were upregulated in response to CC, we observed significant enrichment in structures such as supramolecular fibers, supramolecular polymers, cell junctions, leading edges of cells, and dendrites (65). Phosphorylation-mediated cytoskeletal remodeling is a key regulatory mechanism in the pathogenesis of allergic diseases such as AR and asthma, as it modulates epithelial barrier integrity, immune cell migration, and inflammatory responses, for example, MAP2, P21-activated kinase (PAK), and the actin-related protein 2/3 (Arp2/3) complex.

In our study, we found that PAK, a core regulator of actin cytoskeleton dynamics, is phosphorylated at Ser58 and Ser209. Phosphorylation at these sites activates PAK kinase activity, promoting actin filament polymerization and rearrangement, which disrupts the nasal mucosal epithelial barrier in AR—an essential first line of defense against allergens. Aberrant PAK phosphorylation further enhances the migration and infiltration of eosinophils and mast cells into inflamed tissues, amplifying allergic inflammation (66–68). MAP2, involved in microtubule stabilization, is phosphorylated at Ser283 and Ser608. This modification reduces the affinity of MAP2 for microtubules, impairing the integrity of the microtubule cytoskeleton and compromising immune cell (e.g., T lymphocyte) trafficking (69). The Arp2/3 complex, which is critical for actin filament branching, shows altered phosphorylation at Ser310 and Ser311. Phosphorylation of these subunits enhances Arp2/3-mediated actin network formation, driving airway smooth muscle cell contraction and proliferation in asthma, as well as nasal mucosal edema and hypersecretion in AR (70). This pattern of enrichment strongly indicates that elevated protein phosphorylation primarily serves to maintain cellular morphology, establish cell polarity, and regulate intercellular connections. Such mechanisms may directly contribute to the regulation of immune cell migration and epithelial barrier function during allergic inflammation. This may underlie the critical phenotypic features such as epithelial barrier dysfunction, immune cell infiltration, and tissue remodeling observed in AR (71, 72).

Our network analysis of kinase substrates predicted the involvement of multiple kinases, with a notable subset demonstrating altered activity. Our integrated signaling pathway analysis revealed potential bidirectional regulation within the ERK1/2 pathway. Specifically, our phosphoproteomic data identified concurrent upregulation and downregulation of phosphorylation events, which may reflect cell type-specific responses or feedback mechanisms that fine-tune inflammatory signaling. This intricate regulatory network highlights the complexity inherent in phosphorylation-mediated signaling during allergic inflammation. Our phosphoproteomic data strongly implicate the MAPK/ERK pathway as a pivotal signaling node within the AR nasal mucosa (73, 74). The prioritization of ERK for functional validation was based on multi-dimensional screening criteria. First, kinase activity prediction via motif analysis and substrate enrichment profiling consistently identified ERK1/2 as exhibiting the highest activation score among all differentially regulated kinases. Second, extensive literature on pediatric AR robustly implicates the ERK pathway in Th2 cell differentiation, eosinophilic infiltration, and mucus hypersecretion—key pathophysiological hallmarks of childhood AR—whereas other enriched kinases (e.g., p38 MAPK and JNK) are more broadly associated with generic cellular stress responses and lack well-established, specific links to pediatric nasal remodeling or AR-specific immunopathology. Third, our murine model recapitulates hallmark features of Th2-polarized nasal inflammation, closely aligning with the ERK-dependent immune skewing phenotype previously documented in allergic airway disease. Our results showed that inhibition of ERK signaling in the nasal mucosa alleviated AR symptoms and associated tissue remodeling. Furthermore, the expression levels of a number of cytokines, especially cytokines related to inflammation and immune responses, such as IL-2, IL-4, IL-6, and TNF, were altered after PD98059 treatment (**Figures 7A–D**) (75, 76). Given this prominence, further elucidation of the specific role of the ERK signaling axis in allergic inflammation and its translational potential is warranted. The ERK cascade functions as a critical convergence point that links cell surface receptors to nuclear transcription factors, thereby orchestrating the initiation and maintenance of allergic responses. Existing evidence indicates that the activation of ERK1/2 is indispensable for the differentiation of CD4^+^ T cells into the Th2 subset (77–79). Mechanistically, T-cell receptor (TCR) signaling relies on the ERK pathway, thereby driving the transcription and secretion of prototypical Th2 cytokines, including IL-4, IL-5, and IL-13 (80, 81). This Th2-skewed immunophenotype underlies the pathophysiology of allergic rhinitis (AR) and asthma. Furthermore, at the effector cell level, sustained ERK phosphorylation prolongs eosinophil survival and facilitates mast cell degranulation, releasing inflammatory mediators such as histamine and leukotrienes. ERK activation in nasal epithelial cells has also been demonstrated to regulate barrier integrity and mucin (MUC5AC) hypersecretion (82, 83). Therefore, the ERK pathway functions not only as a driver of immune dysregulation but also as a direct effector, exacerbating tissue remodeling and clinical symptoms. This may provide a potential explanation for the complex phosphorylation dynamics observed in the dataset under study.

In the context of allergic disease, inhibition of the ERK pathway has been shown to have significant anti-inflammatory potential. Preliminary investigations suggest that the administration of particular Mitogen-Activated Protein Kinase (MEK)/ERK inhibitors, such as U0126, in asthma models results in a substantial reduction in eosinophil infiltration within the airway and nasal mucosa (84–86). This intervention has also been shown to decrease serum-specific IgE levels and mitigate airway hyperresponsiveness. Moreover, recent reports have described the development of small molecules capable of selectively disrupting the interaction between ERK and distinct substrates (e.g., c-Fos) while maintaining basal survival functions has emerged as a promising strategy for precisely targeting inflammatory transcriptional programs (87). This approach offers a promising avenue to circumvent the systemic toxicity of broad-spectrum kinase inhibitors in the development of novel antiallergic therapeutics. Although the effects of ERK inhibitors need to be confirmed by further studies, targeting ERK may help prevent nasal inflammation in AR.

Several limitations of this study should be acknowledged. First, our conclusions regarding ERK as a pivotal kinase in AR are derived from a juvenile mouse model; these findings cannot fully establish ERK as the primary upstream driver in human pediatric AR. Second, the pharmacological intervention using PD98059 only verified the partial functional role of ERK in alleviating nasal inflammation, which does not prove that ERK is the exclusive or top-tier upstream trigger of the entire allergic inflammatory network. Future studies employing multi-pathway combined knockout or overexpression approaches are warranted to clarify the hierarchical upstream and downstream relationships among MAPK family kinases in AR pathogenesis. Third, all phosphoproteomic and pharmacological data presented herein were obtained from a 3-week-old juvenile mouse model, which, while recapitulating key features of pediatric AR, does not fully replicate the complexity of human nasal epithelial structure and local immune microenvironment. Interspecies differences must not be overlooked. Therefore, target validation using nasal biopsy specimens or mucosal scrapings obtained from pediatric AR patients represents an essential prerequisite for any clinical translation of ERK-targeted therapeutics. Our team plans to prospectively collect such samples in upcoming studies to validate ERK phosphorylation dynamics in human tissues.

In conclusion, our study aimed to elucidate the molecular mechanisms underlying the nasal mucosa dysfunction induced by AR. To achieve this objective, we conducted phosphoproteomic profiling and constructed a comprehensive and intricate map of phosphorylation within the nasal mucosa. Our analysis revealed 3,861 proteins encompassing a total of 15,491 phosphorylation sites. Specifically, we detected 441 downregulated phosphorylation sites on 584 proteins and 531 upregulated phosphorylation sites on 722 proteins in the nasal mucosa of the AR group. Our proteomics findings suggest that the dysregulation of immune activation and metabolic regulation may contribute to AR pathophysiology. Through pathway analysis of the identified phosphopeptides, we found that ERK signaling emerged as a pivotal converged node mediating OVA-induced nasal inflammatory responses; notably, upregulation of ERK1/2 phosphorylation was identified as a significant marker associated with AR. Importantly, targeting ERK inhibitors presents a potential therapeutic strategy for modulating key inflammatory response signaling pathways in the context of AR, although it represents one of several kinase pathways implicated. These results underscore that understanding the molecular mechanisms driving AR-induced alterations in nasal mucosal function can pave the way for novel treatments for allergy-related diseases. Overall, elucidating these mechanisms has substantial implications for developing targeted interventions aimed at mitigating inflammation associated with allergic rhinitis.

## Data Availability

The datasets presented in this study can be found in online repositories. The names of the repository/repositories and accession number(s) can be found below http://www.proteomexchange.org/, PXD073099.

## Supplementary material

Supplementary Figure 1 Quality control and preliminary characterization of proteomic data. **(A)** Bar chart summarizing the numbers of identified peptides and proteins. **(B)** Heatmap showing Pearson correlation coefficients **(PCC)** among proteomic samples. **(C)** Bar chart showing level-2 GO classification of all quantified proteins.
